# Decreased DNA-PK activity in human cancer cells exhibiting hypersensitivity to low-dose irradiation

**DOI:** 10.1054/bjoc.2000.1258

**Published:** 2000-07-24

**Authors:** S Vaganay-Juéry, C Muller, E Marangoni, B Abdulkarim, E Deutsch, P Lambin, P Calsou, F Eschwege, B Salles, M Joiner, J Bourhis

**Affiliations:** Unité Propre de l’Enseignement Supérieur ‘Radiosensibilité-Radiocarcinogenèse humaine’ (UPRES EA 27–10 Pr. Eschwege), Institut Gustave Roussy, IFR No 54, Villejuif, 94805, France; Institut de Pharmacologie et de Biologie Structurale CNRS, Toulouse, 31077, France; Gray Laboratory Cancer Research Trust Mount Vernon Hospital, Northwood, Midlessex, UK

**Keywords:** ionizing radiation, DNA repair, low-dose hyper-radiosensitivity, DNA-PK

## Abstract

Low-dose hyper-radiosensitivity (HRS) (below 0.5 Gy) has been extensively documented in the past few years. The molecular basis of this phenomenon remains largely unknown and the purpose of this study was to investigate the possible implication of the DNA repair DNA-PK complex. The activity of the DNA-PK complex, i.e. Ku DNA-end binding activity and kinase activity of the whole complex, was studied in 10 human cancer cell lines, 2 h after 0.2, 0.5 and 1 Gy irradiation. After low-dose irradiation (0.2 Gy), a marked decrease in DNA-PK activity was found in all six cell lines exhibiting HRS, whereas the DNA-PK activity was increased in the four cell lines which did not exhibit HRS. This modulation of DNA-PK activity was a rapid phenomenon occurring within the 2 h following low-dose radiation exposure. These data strongly suggest the implication of the DNA-PK repair complex in the HRS phenomenon. © 2000 Cancer Research Campaign


British Journal of Cancer (2000) **83**(4), 514–518
© 2000 Cancer Research Campaign
doi: 10.1054/ bjoc.2000.1258, available online at http://www.idealibrary.com on IDEAL®

### Decreased DNA-PK activity in human cancer cells exhibiting hypersensitivity to low-dose irradiation

**S Vaganay-Juéry[1], C Muller[2], E Marangoni[1], B Abdulkarim[1], E Deutsch[1], P Lambin[3], P Calsou[2], F Eschwege[1], B Salles[2], M Joiner[3] and J Bourhis[1]**

[1]Unité Propre de l'Enseignement Supérieur 'Radiosensibilité-Radiocarcinogenèse humaine' (UPRES EA 27–10, Pr. Eschwege), IFR No 54, Institut Gustave Roussy, Villejuif 94805, France; [2]Institut de Pharmacologie et de Biologie Structurale, CNRS, 31077 Toulouse, France; [3]Gray Laboratory Cancer Research Trust Mount Vernon Hospital, Northwood, Midlessex, UK

**Summary** Low-dose hyper-radiosensitivity (HRS) (below 0.5 Gy) has been extensively documented in the past few years. The molecular basis of this phenomenon remains largely unknown and the purpose of this study was to investigate the possible implication of the DNA repair DNA-PK complex. The activity of the DNA-PK complex, i.e. Ku DNA-end binding activity and kinase activity of the whole complex, was studied in 10 human cancer cell lines, 2 h after 0.2, 0.5 and 1 Gy irradiation. After low-dose irradiation (0.2 Gy), a marked decrease in DNA-PK activity was found in all six cell lines exhibiting HRS, whereas the DNA-PK activity was increased in the four cell lines which did not exhibit HRS. This modulation of DNA-PK activity was a rapid phenomenon occurring within the 2 h following low-dose radiation exposure. These data strongly suggest the implication of the DNA-PK repair complex in the HRS phenomenon. © 2000 Cancer Research Campaign

**Keywords**: ionizing radiation; DNA repair; low-dose hyper-radiosensitivity; DNA-PK

The response to single-dose irradiation is dose-dependent, so that exposures at very low doses (below 0.5 Gy) are generally more effective per unit dose than larger exposures (Figure 1). This phenomenon has been termed low-dose hyper-radiosensitivity (HRS) (Joiner et al, 1996) and has been extensively described. It was initially recognized in vivo in a skin model for acute reaction and further in kidney (Joiner et al, 1996). It has also been reported in protozoa, mammalian and human cells in vitro (Joiner et al, 1996; Marples et al, 1997; Short and Joiner, 1998), and recently by Turesson in normal epithelial tissues from patients with prostate carcinoma (Turesson et al, 1998). This HRS phenomenon is of particular interest in cancer cells since we have shown in a series of more than 25 cancer cell lines that it was more pronounced in the most radioresistant cell lines at higher doses, with only a few exceptions (e.g. SiHa and U373 cell lines) (Lambin et al, 1996; Short et al, 1999; Short, 1999). This hypersensitivity at low doses in cancer cells harbouring radio-resistance at higher doses has prompted several authors to test the value of low-dose irradiation in the clinic, especially in patients with cancers refractory to conventional radiotherapy (M. Saunders, P. Lambin personal communications).

The molecular mechanisms underlying this HRS phenomenon remain largely unknown. Following low-dose ionizing radiation exposure, the transcription of immediate early genes (c-*jun*, c-*fos*, c-*myc*) has been reported (Prasad et al, 1995). However, this induction has not been correlated with the presence of HRS. HRS processes appeared to be related to the amount of repair of radio-induced DNA damage (Marples et al, 1997) rather than a modulation of cell-cycle progression or apoptosis (Power, 1997). It is thus conceivable that some changes in expression and/or activity of proteins involved in DNA double-strand break (DSB) repair might be implicated in this process, since DSB repair has been shown to play an important role in the radiation response of mammalian cells. Indeed, a parallel between radiosensitivity and the yield of unrepaired DSB has been reported in various models (Allalunis-Turner et al, 1995). Of particular interest is the DNA-dependent protein kinase (DNA-PK) complex which plays a major role in DSB repair. It is a serine-threonine protein kinase that requires DSB for activity, and is composed of a large catalytic subunit, the DNA-PKcs, and a smaller DNA-targeting component, the Ku heterodimer (Ku80 and Ku70). The catalytic subunit DNA-PKcs is a protein of 460 kDa which belongs to the phosphatidyl (PI)-3 kinase family (Smith and Jackson, 1999). DNA-PKcs is recruited to DNA by Ku-binding (Gottlieb and Jackson, 1993) and can phosphorylate several transcription factors in vitro, such as *p53*, c-*Jun* and other DNA-binding proteins (Shieh et al, 1998). In rodent cells, the DNA-PK complex has been shown to play a major role in determining radiosensitivity (Jeggo, 1998) and it is likely that the DNA-PK complex also plays a key role in determining the radiosensitivity of human cells, since we have shown recently that an antisense against Ku80 was able to decrease the DNA-PK kinase activity and DSB repair capacity and hence to strongly increase the radiosensitivity of transformed human cells (Marangoni et al, 2000). In this context, the purpose of this study was to investigate the possible implication of DNA-PK in the HRS process. We report here a striking correlation between the existence of HRS and the variation of the DNA-PK complex kinase activity following low-dose irradiation, among 10 human cancer cell lines exhibiting various HRS characteristics.

*Received 23 November 1999*
*Revised 30 March 2000*
*Accepted 3 April 2000*

*Correspondence to*: J Bourhis

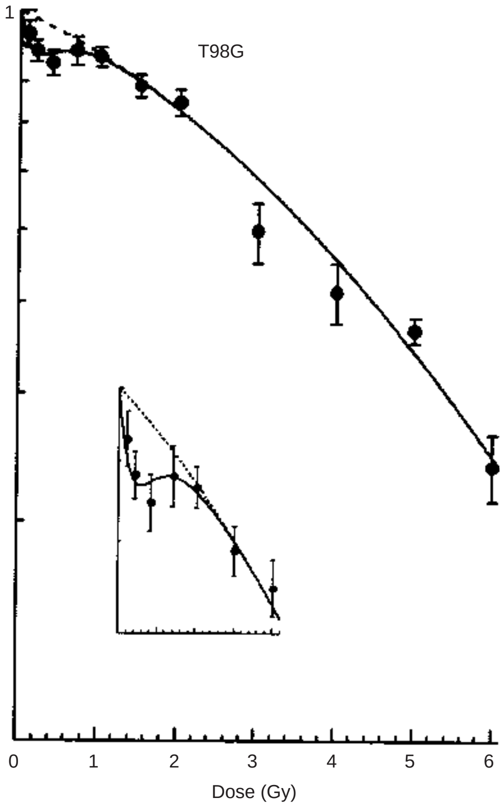

**Figure 1** Dose response in T98G human glioblastoma cells in vitro showing low doses (< 0.5 Gy) hyper-radiosensitivity (Short et al, 1999)

### MATERIALS AND METHODS

### Cell lines

Ten human cancer cell lines were used which have been previously characterized by clonogenic survival assay for their radiation sensitivity at low (< 0.5 Gy) and high doses (> 0.5–2 Gy) (Lambin et al, 1996; Short et al, 1999). The HRS characteristics of the cell lines have been obtained by fluorescence-activated cell sorter (FACS) and dynamic microscope image processing scanner (DMIPS). The HRS+ cell lines were characterized by the 'substructure' of their survival curves below 1 Gy (Figure 1) which deviates from the prediction extrapolated from higher-dose data, using the linear quadratic formula (Short and Joiner, 1998). The distribution of the cell lines according to their radiosensitivity at high doses, and the presence or absence of HRS is reported in Table 1. Cell lines were routinely cultured in Nunclon plastic flasks containing Earle's minimum essential medium supplemented with 15% fetal calf serum, 3 mM glutamine, non-essential amino acids, 100 U $ml^{-1}$ penicillin and 100 μg streptomycin with the exception of HX142 (HamF10 medium) and SiHa (DMEM). Cells were grown as monolayers at 37°C under 5% $CO_2$.

### Irradiation

Irradiation was performed using a $^{137}$Cs γ-ray source at a dose rate of 1.45 Gy $min^{-1}$ as we have described previously (Badie et al, 1995). Briefly, cells were irradiated in exponential phase at different doses (0, 0.2, 0.5, and 1 Gy) and harvested after 2 h at 37°C for cell extracts. A time effect was also studied by analysing DNA-PK activity at various time after irradiation (0, 1, 2, and 6 h after radiation exposure).

### Cell extracts

Approximately $5 \times 10^6$ exponentially growing cells were scraped from the dishes in PBS using a rubber policeman and centrifugated at 4°C for 5 min at 1500 rpm. To make whole cell extracts, cell pellets were resuspended in 2 volumes of lysis buffer 1 (50 mM NaF, 450 mM NaCl, 20 mM Hepes pH 7.8, 25% w/v glycerol, 0.2 mM EDTA, 0.5 mM DTT) in the presence of proteinase inhibitors (Complete, Boerhinger, Germany). The swollen cells were disrupted by incubation alternatively on liquid nitrogen and 30°C (4 times) for 3 min each. The resulting suspension was sedimented by a 30-min centrifugation at 13 000 rpm and 4°C and the supernatant removed and aliquoted.

### DNA-PK assay

A DNA-PK 'pulldown' kinase assay was performed as described previously (Finnie et al, 1995). This assay contains a purification on double-stranded DNA cellulose beads which retains only proteins able to bind DNA. Each sample was assayed in the presence of either DNA-PK-specific peptide substrate (SQE

**Table 1** Distribution of the cell lines according to their radiosensitivity at high doses, as shown by the surviving fraction at 2 Gy (SF2) and their sensitivity at low doses (presence or absence of low-dose hyper-radiosensitivity = HRS+ and HRS–, respectively)

| | Radioresistant cell-lines SF2>50% | | | Radiosensitive cell-lines SF2<50% | | |
|---|---|---|---|---|---|---|
| | | SF2 (%) | Baseline DNA-PK activity[a] | | SF2 (%) | Baseline DNA-PK activity[a] |
| HRS+ | HgL21 | 60 | 500 | MeWo | 29 | 700 |
| | RT112 | 62 | 1600 | | | |
| | T98G | 63 | 400 | | | |
| | Bell | 68 | 950 | | | |
| | HT29 | 74 | 2000 | | | |
| HRS– | U373 | 55 | 700 | SW48 | 18 | 2300 |
| | SiHa | 64 | 700 | HX142 | 4 | 260 |

[a]The basal level (in the absence of irradiation) of DNA-PK activity is expressed in cpm $\mu g^{-1}$ of protein extracts

© 2000 Cancer Research Campaign peptide: EPPL**SQE**AFADLLKK) or a negative control peptide (SEQ peptide: EPPL**SEQ**AFADLLKK). DNA-PK activity was expressed in cpm incorporated in the SQE peptide or in the SEQ peptide for a given extract. In additional experiments, after incubation of the extracts with double-stranded DNA cellulose beads (Sigma, France), beads were extensively washed with kinase assay Z' buffer (Finnie et al, 1995) and electrophoresed in a 10% SDS-polyacrylamide gel as we have described previously (Muller et al, 1998). Samples were analysed by Western blotting as described below for the presence of DNA-PKcs and Ku proteins.

### Band shift assay

In human cell lines, Ku represents the major double-strand (ds) DNA end-binding protein and its activity can be easily detected by using dsDNA fragments in an electrophoretic mobility shift assay (EMSA).

The double-stranded 14-mer DNA (5´GGGCCCGGGACG-CG3´) was radiolabeled with $\gamma$-$^{32}$P-ATP using T4 polynucleotide kinase (Biolabs) and ends-labelled probes were separated from unincorporated nucleotides by Chroma spin-10 column (Clontech Laboratories). For band shift assay, 0.1 ng of $^{32}$P-labelled DNA (100 000 cpm) was incubated with 2.5 µg of cell extracts and 500 ng of closed circular plasmid DNA as a non-specific competitor in 20 µl of binding buffer (100 mM Tris-HCl, pH 7.5, 0.5 mM EDTA, 25% glycerol, 10 mM $MgCl_2$, 1.25 mM DTT) at 30°C for 10 min. The samples were electrophoresed on a 4% polyacrylamide gel at 4°C for 2 h at 100 V. The gel was dried on Whatman paper and exposed to film.

### Western blotting

50 µg cell extracts were boiled in SDS-PAGE loading buffer and separated by 10% SDS-PAGE. The protein transfer to nitrocellulose was performed with a West-blotting system and blocked for 2 h in TBS 5% milk solution. Thereafter, Ku-86 monoclonal antibody (Mab11, Interchim, France) was diluted 1:1000 overnight in TBS 0.1% Tween20. The same procedure was applied using monoclonal antibodies directed against Ku70 (1:1000, N3H10, Interchim, France) and DNA-PKcs (1:1000, 18–2 gift from Dr T Carter, St John's University, New York or 1:200, sc-1551, Santa Cruz). After washing for 1 h, the secondary anti-mouse antibody diluted at 1:5000 was applied to the membrane and then rewashed. Blotting was revealed using a Pierce kit, ECL kit (Amersham, Buckinghamshire, U.K.).

### RESULTS

We investigated the activity of the DNA-PK complex, i.e. Ku DNA-end-binding activity and kinase activity of the whole complex, in 10 human cancer cell lines. The DNA-PK complex kinase activity was evaluated 2 h after 0.2, 0.5 and 1 Gy. A marked decrease in the activity was evident after 0.2 Gy in six cell lines whereas in the remaining four cases the DNA-PK activity was increased. Strikingly, a decrease of the DNA-PK activity was only found in the six cell lines exhibiting HRS (range 24–40%), whereas the DNA-PK activity markedly increased (range 20–40%) in the four cell lines which did not exhibit HRS (Figure 2). After2 h and 0.5 Gy, in most cell lines exhibiting the HRS phenomenon, a decrease in DNA-PK activity was also observed, except for Hg121 cells, whereas the DNA-PK activity increased in cell lines with no HRS, except for HX142. After 1 Gy no correlation was found (data not shown).

We further investigated whether the DNA-PK kinase activity could vary according to the time after irradiation. The T98G and HT29 cell lines were chosen for studying the time course of the variations of DNA-PK activity after low-dose irradiation. For both cell lines, the maximum decrease of DNA-PK activity was found before 2 h after irradiation (Figure 3).

We then investigated the molecular mechanisms underlying the observed variations of the DNA-PK activity after low-dose irradiation exposure. As Ku DNA-binding represents the predominant mechanism for DNA-PK activation (Finnie et al, 1995), we first studied the Ku DNA-end-binding activity in HRS+ and HRS– cell lines. We were not able to detect significant variations within 2 h after exposure to low-dose irradiation, as reported in Figure 4. This suggests that the variations of the whole DNA-PK complex activity was not due to a change in Ku DNA-end-binding capacity.

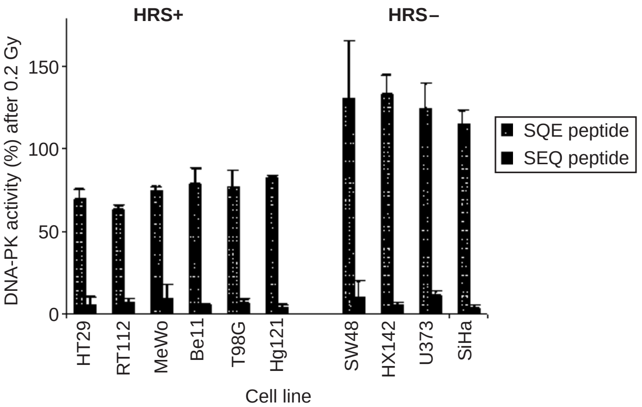

**Figure 2** Relative variation (%) of the DNA-PK kinase activity, between 0 Gy and 0.2 Gy, 2 h after irradiation, for the six cell lines exhibiting low-dose hyperradiosensitivity (HRS+) and the four cell lines which do not exhibit low-dose hyperradiosensitivity (HRS–). The control value on SEQ peptide is representative of the DNA-PK phosphorylation specificity. The DNA-PK basal activity (without irradiation) is arbitrarily normalized to 100%. Error bars are obtained from at least three experiments

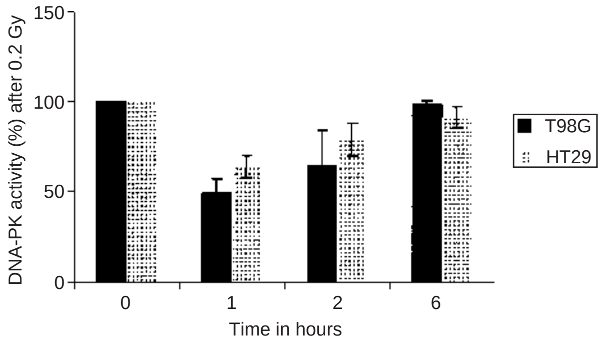

**Figure 3** Influence of the time (0–6 h) after 0.2 Gy exposure on DNA-PK kinase activity (expressed as the % of the basal level in the absence of irradiation), for T98G cells and HT29 cells. Error bars are obtained from three experiments

*© 2000 Cancer Research Campaign*

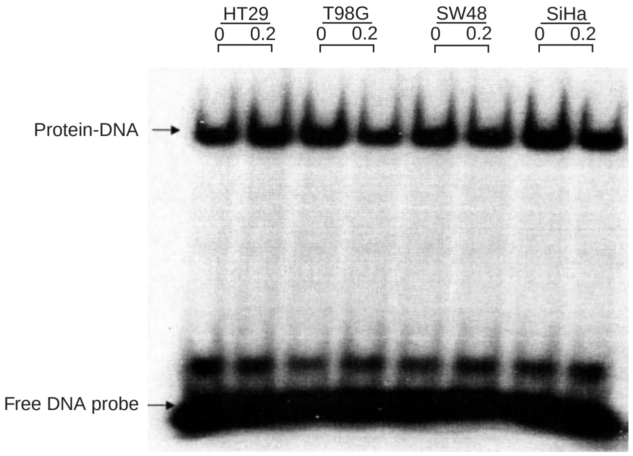

**Figure 4** Ku DNA-ends binding activity of extracts from control and from 0.2 Gy-irradiated cells after 2 h. Two HRS+ cell lines (HT29 and T98G) and two HRS– cell lines (SW48 and SiHa) are presented

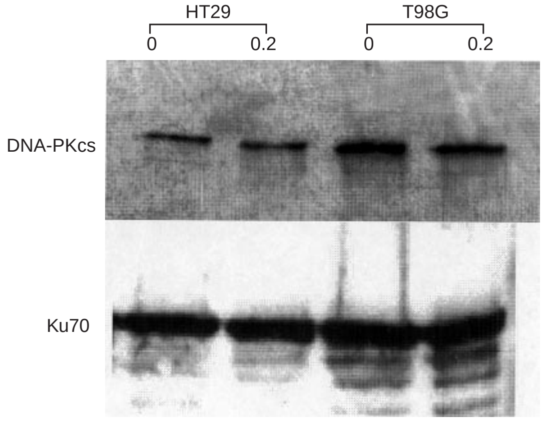

**Figure 6** Expression of Ku70 and DNA-PKcs by Western immunoblotting 2 h after 0 or 0.2 Gy and purification DNA cellulose beads, for HT29 and T98G cell lines. The beads contain DNA fragments and allow exclusive purification of proteins able to bind on DNA ends (see Materials and methods)

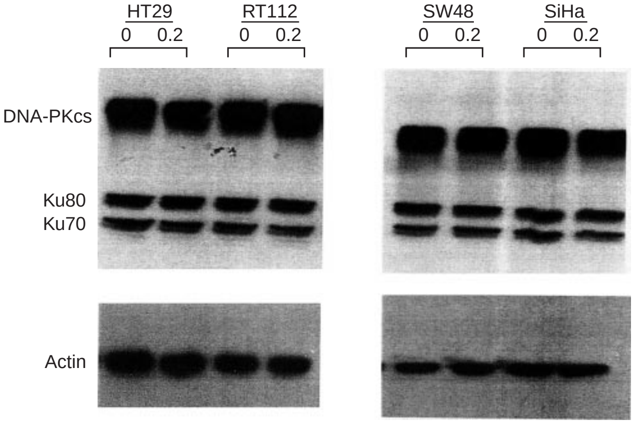

**Figure 5** Expression of Ku80, Ku70 and DNA-PKcs by Western immunoblotting 2 h after 0 or 0.2 Gy in four different cell lines. Two HRS+ cell lines (HT29 and RT112) and two HRS– cell lines (SW48 and SiHa) are presented

We also analysed in all the 10 cell lines the level of expression of the three components of the DNA-PK complex, i.e. Ku70, Ku80 and DNA-PK by Western blotting. No significant variations of these three proteins was found 2 h after radiation exposure, indicating that the changes in DNA-PK activity were not associated with modifications of the protein levels (Figure 5). Finally, we explored whether Ku heterodimer was able to recruit the catalytic subunit on DNA ends. To test this hypothesis, the proteins associated to double-stranded DNA-cellulose beads were eluted and subjected to Western blot analyses. Beads which had been incubated either with control or irradiated protein extracts contained DNA-PKcs and Ku70 (Figure 6). This result suggests that DNA-PKcs is still recruited by Ku complex after 0.2 Gy.

### DISCUSSION

In this study, we observed that the six cell lines exhibiting HRS showed a DNA-PK activity decrease, 2 h after 0.2 Gy irradiation, which was not found in the four HRS− cell lines. Large variations of DNA-PK activity basal levels were found between cell lines (Table 1), without any correlation with clonogenic survival at 2 Gy (SF2), as well with the presence of HRS. This is in good agreement with the recent study published by Kasten et al (1999), showing the absence of correlation between DNA-PK activity and SF2.

The analysis of the DNA-PK complex activity 2 h after 0.2 Gy revealed a strong correlation with the presence of HRS. Doses of 0.5 Gy and higher were also tested since the HRS was known to be detectable up to 0.5 Gy, but not at higher doses. The correlation between the variations of the DNA-PK complex activity and the presence of HRS decreased as the dose increased above 0.5 Gy (data not shown). Furthermore, the data we obtained after exposure to 1 Gy are in good agreement with previous studies showing that there was no evidence that irradiation (at high dose) could modulate DNA-PK activity (Lees Miller et al, 1995; Jongmans et al, 1996). In contrast, the present study is the first to show the possible regulation of DNA-PK activity by ionizing radiation, but this was observed exclusively after exposure to low doses.

Importantly, time-course experiments showed that the variations of the DNA-PK activity after low-dose irradiation was a rapid phenomenon occurring within the first few hours following radiation exposure. Although the variations magnitude of the DNA-PK activity were relatively moderate, it is likely that they can have an influence on radiosensitivity. Indeed, the transient decrease of DNA-PK activity within the first hours following low-dose irradiation could contribute to increase of the amount of unrepaired damage, since most of the repair and enzymatic mechanisms generally occur within the first hours following the radiation exposure (Foray et al, 1997). In addition, our results are in agreement with our previous experiments showing that a Ku80 mRNA antisense, which was able to decrease the DNA-PK activity by 50%, was able to induce a major increase in the radiosensitivity of human transformed cells, along with 20% of unrepaired DSB 24 h after irradiation (Marangoni et al, 2000). Given these data, it is conceivable that the decrease of DNA-PK activity we observed after low doses in the HRS+ cell lines may impact radiosensitivity

*© 2000 Cancer Research Campaign*

and DSB repair. Indeed this decrease was observed within the 2 h following irradiation corresponding to a ‘time window’ of maximal DSB repair (Foray et al, 1997).

The catalytic subunit of the complex, DNA-PKcs, is known to be an early marker of apoptosis as this protein is cleaved during the apoptotic process (Song et al, 1996). As reported in Figure 5, there was no suggestion (in any of the cell lines) of induced apoptosis after low doses irradiation, since the band intensity corresponding to the DNA-PKcs did not change after 0.2 Gy. This is in agreement with previous hypothesis reported by Marples et al (1997) and Power (1997) who suggested that DNA repair was involved in HRS rather than apoptosis.

We couldn’t detect any significant change in DNA-PKcs, Ku80 and Ku70 protein levels, protein–protein interactions and DNA-binding capacity of the DNA-PK complex after low-dose irradiation, but the ‘blot quantification’ we performed (data not shown) was perhaps not sensitive enough to illustrate differences such as those we observed with DNA-PK activity measurements. However the DNA-PK activity variation may occur by another channel and it should be pointed out that the mechanisms underlying the regulation of the DNA-PK activity are poorly understood. It is thus possible that the observed variations of the DNA-PK kinase activity might be due to changes in the phosphorylation state, which could involve DNA-PKcs autophosphorylation (Chan et al, 1996). In addition, the c-*abl* protein tyrosine kinase, which is known to be activated by ionizing radiation, has been reported to phosphorylate DNA-PK in vitro and in vivo and to bind with Ku70 (Kharbanda et al, 1997) and could also be responsible for the changes of DNA-PK activity after low-dose irradiation. However, it has been shown that the phosphorylation of DNA-PK by c-*abl*, as well as its autophosphorylation, inhibits the ability of DNA-PKcs to bind to Ku-DNA complex, which was not observed in our study. Indeed, as shown in Figure 6, the variations of DNA-PK activity were not due to a decrease in the fixation of DNA-PKcs on Ku and DNA-ends.

In conclusion, the DNA-PK complex was targeted because of its major role in determining radiation sensitivity, via the involvement in DSB repair both in rodent and human cells. Our data obtained in 10 human cancer cell lines strongly suggest that rapid changes in DNA-PK activity, occurring within few hours after radiation exposure, could be involved in low-dose hyper-radiosensitivity.

### ACKNOWLEDGEMENTS

This work was supported in part by Ligue Nationale Contre le Cancer, Association pour la Recherche sur le Cancer ARC 96–92, Institut Fédératif de Recherche, IFR No 54, Institut Gustave Roussy (Bourse ‘Project IFR’, 1998–99). We gratefully thank Nicolas Foray PhD for discussions.

### REFERENCES

Allalunis-Turner MJ, Zia PKY, Barron GM, Mirzayans R and Day III RS (1995) Radiation-induced DNA damage repair in cells of a radiosensitive human malignant glioma cell line. *Radiat Res* **144**: 288–293

Badie C, Iliakis G and Foray N (1995) Induction and rejoining of DNA double-strand breaks and interphase chromosome breaks after exposure to X-rays in one normal and two hypersensitive human fibroblast cell lines. *Radiat Res* **144**: 26–35

Chan DW and Lees-Miller SP (1996) The DNA-dependent protein kinase is inactivated by autophosphorylation of the catalytic subunit. *J Biol Chem* **89**: 36–41

Finnie NJ, Gottlieb TM, Blunt T, Jeggo PA and Jackson SP (1995) DNA-dependent protein kinase activity is absent in xrs-6 cells: implications for site-specific recombination and DNA double-stand break repair. *Proc Natl Acad Sci USA* **92**: 320–324

Foray N, Priestley A, Alsbeih G, Badie C, Capulas EP, Arlett CF and Malaise EP (1997) Hypersensitivity of ataxia telangiectasia fibroblasts to ionizing radiation is associated with a repair deficiency of DNA double-strand breaks. *Int J Radiat Biol* **72**: 271–283

Gottlieb TM and Jackson SP (1993) The DNA-dependent protein kinase requirement for DNA ends and association with Ku antigen. *Cell* **72**: 131–142

Jeggo PA (1998) Identification of genes involved in repair of DNA double strand breaks in mammalian cells. *Radiat Res* **150**: S80–91

Joiner MC, Lambin P, Malaise EP, Robson T, Arrand JE, Skov KA and Marples B (1996) Hypersensitivity to very-low single radiation doses: its relationship to adaptative response and induced radioresistance. *Mutat Res* **358**: 171–183

Jongmans W, Artuso M, Vuillaume M, Bresil H, Jackson SP and Hall J (1996) The role of Ataxia telangiectasia and the DNA-dependent protein kinase in the p53-mediated cellular response to ionising radiation. *Oncogene* **13**: 1133–1138

Kasten U, Plottner N, Johansen J, Overgaard J and Dikomey E (1999) Ku70/80 gene expression and DNA-dependent protein kinase (DNA-PK) activity do not correlate with double-strand break (dsb) repair capacity and cellular radiosensitivity in normal human fibroblasts. *Br J Cancer* **79**: 1037–1041

Kharbanda S, Pandey P, Jin S, Inoue S, Bharti A, Yuan ZM, Weichselbaum R, Weaver D and Kufe D (1997) Functional interaction between DNA-PK and c-Abl in response to DNA damage. *Nature* **386**: 732–735

Lambin P, Malaise EP and Joiner MC (1996) Might intrinsic radioresistance of human tumour cells be induced by radiation? *Int J Radiat Biol* **69**: 279–290

Lees-Miller SP, Godbout R, Chan DW, Weinfeld M, Day RS 3rd, Barron GM and Allalunis-Turner J (1995) Absence of p350 subunit of DNA-activated protein kinase from a radiosensitive human cell line. *Science* **267**: 1183–1185

Marangoni E, Le Romancer M, Foray N, Muller C, Douc Rasy S, Barrois M, Calsou P, Bernier J, Eschwege F, Salles B and Bourhis J (2000) The transfer of an mRNA antisense of Ku86 increases the radiosensitivity of human transformed fibroblasts. *Cancer Gene Ther*, in press

Marples B, Lambin P, Skov KA and Joiner MC (1997) Low dose hyperradiosensitivity and increased radioresistance in mammalian cells. *Int J Radiat Biol* **71**: 721–735

Muller C, Dusseau C, Calsou P and Salles B (1998) Human normal peripheral blood B-lymphocytes are deficient in DNA-dependent protein kinase activity due to the expression of a variant form of the Ku86 protein. *Oncogene* **16**: 1553–1560

Power MO (1997) PhD Thesis, University of London

Prasad AV, Mohan N, Chandrasekar B and Meltz ML (1995) Induction of transcription of ‘immediate early genes’ by low-dose ionizing radiation. *Radiat Res* **143**: 263–272

Shieh SY, Ikeda M, Taya Y and Prives C (1998) DNA damage-induced phosphorylation of p53 alleviates inhibition by MDM2. *Cell* **91**: 325–334

Short SC and Joiner MC (1998) Cellular response to low-dose irradiation. *Clin Oncol* **10**: 73–77

Short SC, Mayes C, Woodcock M, Johns H and Joiner MC (1999) Low dose hypersensitivity in the T98G human glioblastoma cell line. *Int J Radiat Biol* **75**: 847–855

Short SC (1999) PhD Thesis, University of London

Smith GCM and Jackson SP (1999) The DNA-dependent protein kinase. *Genes Dev* **13**: 916–934

Song Q, Lees-Miller SP, Kumar S, Zhang Z, Chan DW, Smith GC, Jackson SP, Alnemri ES, Litwack G, Khanna KK and Lavin MF (1996) DNA-dependent protein kinase catalytic subunit: a target for an ICE-like protease in apoptosis. *EMBO J* **15**: 3238–3246

Turesson Johansson KA, Nyman J, Flogegard M and Wahlgren T (1998) A clinical study on the effect of low dose per fraction. *Radiother Oncol* **48**: S4–15

© 2000 Cancer Research Campaign
